# Examining the Impact of Maternal Individual Features on Children’s Behavioral Problems in Adoptive Families: The Role of Maternal Temperament and Neurobiological Markers

**DOI:** 10.3390/ijerph15020196

**Published:** 2018-01-24

**Authors:** Yagmur Ozturk, Virginia Barone, Lavinia Barone

**Affiliations:** 1 Department of Brain and Behavioral Sciences-DBBS, University of Pavia, 27100 Pavia, Italy; ozturk.yagmur@unipv.it; 2 Department of Molecular and Developmental Medicine, University of Siena, 53100 Siena, Italy; virginia.barone@unisi.it

**Keywords:** parenting, dopamine, temperament, behavioral problems, adoption

## Abstract

The first year after adoption constitutes a sensitive period for both strengthening the new emotional bond in the family and checking its appropriate development by adoption services. A key variable for children’s catch-up are adoptive parents’ socioemotional and individual features. The aim of this study is to investigate links between adoptive mothers’ individual features and behavioral problems in their children in the first year after adoption placement, by testing the moderating role of both age at adoption and maternal genetic polymorphisms. Seventy-eight adoptive mothers completed temperament and genetic measures. Mothers showed a specific pattern of interaction between basic temperament traits and genetic markers in their assessment of children’s behavioral problems; dopamine D4 receptor gene and children’s age at adoption are two moderators in the association in which mothers’ temperament was affecting the evaluation of their children’s behavioral problems. Findings highlight a still undervalued area of parenting resources in the process of post-institutionalized children’s catch-up after adoption placement, by showing how individual features count in the commonly measured variable of children’s behavioral and emotional problems. This could help in orienting identification and choice of key variables for family assessment after adoption placement, thus contributing in fostering children’s healthy development.

## 1. Introduction

Institutionalization is the more common background of internationally adopted children, with consistent research showing how institutional care leads to a number of developmental shortcomings [1,2], with potential lifelong consequences [3]. Particularly, research findings show that post-institutionalized children are at higher risk for behavior problems [4,5]. This is particularly true for late adopted children, i.e., for children who entered a new family after their first year of age, having thus spent more time in institution. In line with the aforementioned evidences, it is not surprising that parents of adoptees report their children as showing more emotional and behavioral problems than parent-reared, non-institutionalized children [6,7]. 

A key variable for children’s catch-up are adoptive parents’ socioemotional [8] and individual features; however, little research has specifically addressed the latter, being mostly focused on the former. The question not yet addressed pertains to factors which may affect parents’ reports of their adoptive children’s behavioral problems. In particular, some parents’ individual features (i.e., temperament traits and genetic markers) and some children’s at risk variables, i.e., age at adoption [9,10] may play a role in the association with one of the main features of children’s mental health, i.e., emotional and behavioral problems. Temperament has been associated with the self-regulatory processes parents use to regulate interaction with their children and select parental strategies they rely on to control their children’s behavior [11]. Negative Affectivity (reactivity process reflecting a general tendency toward experience negative emotions) and Effortful Control (self-regulation process includes inhibitory control and attentional focusing) are two broad constructs of several temperament dimensions [12].

A growing body of literature focuses on susceptibility effects for several genes, including serotonin- and dopamine-related gene polymorphisms, in a “for better or for worse” manner: individuals who are more vulnerable to adverse social environments also benefit more from positive environments [13,14,15]. However, few studies have examined genetic differences interaction with the environment related to parenting [16]. Given the promising outcomes of this research field, the current study focused on the two genetic markers of adoptive mothers, mainly considered in family studies: the dopamine D4 receptor gene (DRD4) and the serotonin transporter gene (5-HTTLPR). The DRD4 gene and 5-HTTLPR gene polymorphisms play a key role in the etiology of attention deficit and hyperactivity disorder [17] and depressive disorder [18] respectively.

The first year after adoption placement represents a key period for acknowledging possible strengths and vulnerabilities of all adopted children and it usually serves as a point of reference for the monitoring work of adoption services that are in charge of checking the new family’s socioemotional adjustment. Children who were adopted before 12 months of age, named early adoptees, were almost as securely attached as their non-adopted peers [19], with related less emotional and behavioral problems, whereas late adopted children appear to be the more at risk group for behavioral problems development.

In view of all mentioned so far, the objective of this research is to investigate links between adoptive mothers’ individual features, including basic temperament traits (negative affectivity and effortful control) and genetic markers (i.e., dopamine and serotonin gene polymorphisms), and maternal report of emotional and behavioral problems in their children in the first year after adoption placement, by testing the moderating role of both age at adoption and maternal genetic polymorphisms (mothers’ DRD4 7-repeat allele and 5HTTLPR short allele). We expect that maternal higher effortful control will be associated with less emotional and behavioral problems in children; and conversely that lower maternal negative affect will be associated with less behavioral problems in the children. Moreover, we hypothesize that children’s age at adoption and maternal genetic polymorphisms could play a moderator role in these relationships, regardless of children’s temperament traits.

## 2. Materials and Methods

### 2.1. Participants

Eighty-one mothers with their first adopted children have been recruited through adoption services. Due to the incomplete data of 3 families, 78 adoptive families were considered. Table 1 shows both mother’s and children’s demographic characteristics. Given the high correlation between age at adoption and age at assessment (*r* = 0.96, *p* < 0.001), we choose to consider the first one as key variable [19]. All children had been internationally adopted and spent their previous life in institution, with no additional available information concerning their earlier experiences.

All subjects gave their informed consent for inclusion before they participated in the study. The study was conducted in accordance with the Declaration of Helsinki, and the protocol was approved by the Ethics Committee of the Department of Psychology (currently Brain and Behavioral Sciences Department) of the University of Pavia (Project identification code: 1-2013).

### 2.2. Measures

*The Child Behavior Checklist for Ages 1.5–5 (CBCL/1.5–5)* [20,21]. Mothers completed a 100-item questionnaire of problem behaviors of preschoolers (18 months through 5 years), rating the frequency of behaviors on a 3-point Likert scale, from 0 (not true) to 2 (very true or often true). The raw scale scores of total problem (CBCL-Total) were used in the present study. Internal consistency was tested with Cronbach’s alpha value (Total problem score = 0.94).

*The Adult Temperament Questionnaire-short form (ATQ)* [22]. Mothers completed a 77-item self-report questionnaire assessing their temperament using a 7-point rating scale. Based on our hypothesis, the Effortful Control (EC) and the Negative Affect (NA) factors were used in our analysis. Cronbach’s alpha values for the current sample were adequate; respectively, α = 0.80 for the NA factor and 0.84 for the EC factor.

*The Child Behavior Questionnaire (CBQ)* [23]. Mothers completed a parent-report measure for children from 3 to 7 years of age to assess children’s temperament using a 7-point Likert-style rating scale (1 = extremely untrue and 7 = extremely true). We used two domains: Negative Affect (CBQ NA) and Effortful Control (CBQ EC). The Cronbach’s alphas were 0.74 for the CBQ NA, and 0.80 for the CBQ EC.

*Neurobiological markers*. Genotyping was conducted at Padua, BMR Genomics lab, using well-established methods. DNA samples were extracted from buccal swabs using the kit IQ form Promega following manufacturer’s instructions. For genotyping the VNTR in exon 3 of the DRD4 gene, PCR was performed using the primers 5′-AGGACCCTCATGGCCTTG HEX-conjugated and 5′GCTCATGCTGCTGCTCTACT were used. PCR was performed as follows: 1 cycle of 5 min at 94 °C, followed by 10 cycles of 45 s at 94 °C, 30 s at 62 °C, 1 min 68 °C, 25 cycles of 30 s 94 °C, 30 s 52 °C, 1 min 68 °C and a final extension step of 7 min at 68 °C. The PCR-amplified DNA fragments were analyzed by loading the PCR product on an ABI PRISM 3130xl Genetic Analyzer.

For amplification of the 5′ regulatory region of Serotonin Transporter gene (Locus Symbol SLC6A4), which contains a 43 bp insertion/deletion polymorphism (5HTTLPR), primers 5′-TCCTCCGCTTTGGCGCCTCTTCC 6FAM-conjugated and 5′-TGGGGGTTGCAGGGGAGATCCTG were used. PCR was carried out as follows: 1 cycle of 5 min at 94 °C, followed by 10 cycles of 45 s at 94 °C, 1 min 68 °C, 1 min 68 °C, 25 cycles of 45 s 94 °C, 45 s 55 °C, 1 min 68 °C and a final extension step of 7 min at 68 °C. The presence of the Short (S) and Long (L) allele for each sample was determined by loading the PCR product on an ABI PRISM 3130xl Genetic Analyzer. All the PCR were carried out in presence of 0.05% DMSO and 1 N betaine starting from 2 µL l of genomic DNA.

### 2.3. Procedure

Mothers were recruited by consecutive admission to adoption services in Italy and were asked, after agreement and signature of informed consent, for completing a demographic information form and the three questionnaires mentioned above. Regarding DNA collection, buccal swabs were collected at the same time of self-report administration and stored at −20 °C until DNA was extracted. The research was part of a more comprehensive intervention trial and was approved by the ethical committee of Pavia University Department of Brain and Behavioral Sciences.

## 3. Results

As all variables were normally distributed, the study hypotheses were tested via parametric analyses. T-scores of CBCL-Total showed that mothers reported 11.5% of children as in the clinical range, 9% of them as in the borderline range, confirming previous findings showing a higher percentage of emotional and behavioral problems among these children, if compared to those reared in biological families. Moreover, there were no significant differences between girls and boys on any of outcome measures (all *p* > 0.05).

### 3.1. Are Mothers’ Basic Temperament Traits Associated with Childrens’ Emotional and Behavioral Problems?

Zero-order correlations analyses showed the association between mothers’ temperamental features and their children’s behavioral problems (NA: *r* = 0.26, *p* < 0.05; EC: *r* = −0.25, *p* < 0.05; see Table 2). Age at adoption was also correlated with children’s behavioral problems, *r* = 0.22, *p* < 0.05.

### 3.2. Do Maternal Genetic Markers and Children’s Age at Adoption Count in This Relationship?

**Figure 1:**
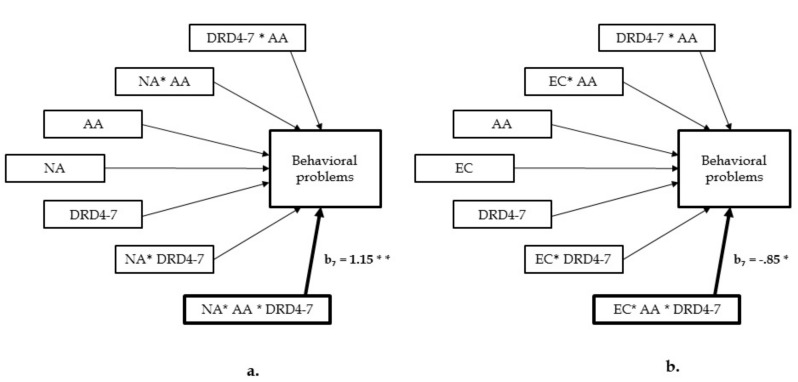
A moderation model with 2 moderators, showing significant effects of 3-way interaction with (**a**) mothers’ temperamental negative affectivity and (**b**) mothers’ temperamental effortful control on the outcome (behavioral problems of children). *b* = unstandardized coefficient; NA = Negative Affect; EC = Effortful Control; AA = Age at adoption; DRD4-7 = the 7r allele of the dopamine D4 receptor gene. * *p* < 0.05, ** *p* < 0.01.

Then, we performed moderation analyses using PROCESS [24,25] to test whether children’s age at adoption and mothers’ genetic markers moderated the effect of mothers’ temperamental features on their children’s behavioral problems. The analyses were carried separately for each independent variable (NA and EC) and also for each genetic marker (DRD4-VNTR and 5HTTLPR). We included genetic markers as moderators as a theoretically chosen variable [16]. As predicted, the overall the model (*R*^2^ = 0.29, *F*(7, 70) = 4.06, *p* < 0.001) and the interaction were significant (*b* = 1.15, *t* = 3.15, *p* = 0.002): Adoptive mothers with temperamental NA and being carrier of DRD4-7 repeat allele, significantly assessed their children as showing more emotional and behavioral problems (age at adoption as high: *b* = 33.36, *t* = 3.99, *p* < 0.001). Conversely, adoptive mothers with temperamental EC and being carriers of DRD4-7 repeat allele significantly assessed their children as showing less emotional and behavioral problems (Overall model: *R*^2^ = 0.21, *F*(7, 70) = 2.57, *p* = 0.02; interaction: *b* = −0.85, *t* = −2.11, *p* = 0.03; age at adoption as high: *b* = −13.72, *t* = −2.08, *p* = 0.04). The models to test the moderation effect of 5HTTLPR was not significant (The model with NA: *R*^2^ = 0.17, *F*(7, 70) = 2.01, *p* = 0.07; EC: *R*^2^ = 0.14, *F*(7, 70) = 1.57, *p* = 0.15) (Figure 1).

### 3.3. Do Temperament Features of Children Count in This Relationship?

Lastly, in order to check if even children’s temperament features could count in the findings obtained, we examined whether both children’s negative affect and effortful control moderated the relationship between mothers’ temperament and children’s behavioral problems (on the sub-sample of older ones *N* = 53). We did not observe any moderator role of children’s temperamental features neither for the model with mothers’ NA (interaction: *b* = −1.9, *t* = −0.32, *p* = 0.74) nor for the model with mothers’ EC (interaction: *b* = −6.5, *t* = −0.90, *p* = 0.37).

## 4. Discussion

Our main goals were to identify first the effect of individual maternal features (e.g., specific temperament traits) on late adopted children’s emotional and behavioral problems and second to inquire how children’s age at adoption placement and maternal genetic polymorphisms influenced this relationship. Lastly, we also checked if children’s temperament features could count. The findings showed that adoptive mothers tended to report more behavioral problems of their children according to specific individual features; mothers who showed more temperamental features of negative affectivity-NA (more sensitivity to a broad spectrum of negative stimuli, fear, anxiety and sadness, depression and aggravation, and frustration), and who were also carriers of dopamine 7r allele of DRD4, showed the highest evaluation of the presence of behavioral problems in their just adopted children. This outcome applies to a specific group of children, i.e., to children whose age at adoption was in the older range considered in our sample—that is about 51 months of age. Conversely, mothers who showed more temperamental effortful control-EC (more self-control, willpower, self-regulation and ability to resolve conflict by inhibiting a dominant response in order to perform a non-dominant response) and who were also carriers of the 7r allele of DRD4 tended to report less behavioral problems, especially for children who arrived in their family older. This result revealed how effective it could be to carefully analyze possible moderators of the association between mothers’ individual features and children’s behavioral problems in order to detect effects that otherwise would remain masked and, thus, undervalued. The interaction between temperamental features and neurobiological markers appears a key concept for identifying the “more affected” group of mothers’ perceptions, “for better and for worse”; the same neurobiological marker, i.e., the dopamine 7r allele of DRD4, appears to play a role in decreasing their children’s behavioral problems report if in interaction with the temperamental feature of mother’s EC and, conversely, in increasing the same perception if in interaction with mother’s NA [15]. Worthy of note, this is especially true for the most challenging children, that is those arrived in the new family at an older age, i.e., after their first year of age.

Outcomes found seem also to enlarge the long history of research on the interaction between parenting and temperament by considering whether, how, and how much parents’ perception of their children’s problem behaviors is influenced in the very demanding situation of the first year after adoption placement [26]. Even if no effect was found for the serotonin moderator, we feel confident that our first results could receive further evidence corroborating or rejecting our pilot inquiry.

In our additional analysis, we did not find any significant role of children’s temperament on how maternal temperament influenced children’s behavioral problems. The absence of an effect is a first pilot indication and it needs to take into account even the size of the sub-sample of older children considered (*N* = 53). Future studies with a bigger sample size could contribute in confirming or rejecting this first finding.

Summing up our main results we can state that the study highlighted a possible undervalued aspect of parental individual differences in a very challenging period as it is the first year after adoption placement. To the best of our knowledge this is the first study trying to look at differences between adoptive mothers by inquiring possible individual factors—like temperamental traits and neurobiological markers—affecting their report on children’s behavior and emotional problems. Given that the constructs inquired appear to be central in understanding the initial attitudes of parents towards their children at the very first stages of the new bond growing up in the adoptive family, our results shed light on a key aspect of this family typology well-being and/or unhealthy functioning. Considering the amount of evidence given on the interplay between attachment and temperament and the current view that conceives the two domains to be permeable one to the other [27], we can speculate on the importance of focusing even on parents’ temperament features as means to inquire their different susceptibility to the environmental cues coming from the recently arrived adoptive child, as a function of individual constitution [17]. Finally, results obtained could be linked to intervention planning, as highlighted in a recent review analyzing costs and savings in parenting interventions [28].

Next to strengths, our study has some limitations, which also constitute future research directions. First, although our sample was large for the population targeted, a larger size could allow a more reliable analysis of the neurobiological moderator effect, possibly including even other “susceptible genes”. Second, fathers could in the future become a target of this kind of inquiry, as their role is increasingly considered in the literature on adoptive parenting [29,30]. Furthermore, this study did not examine the physical health of the children in the adoptive family. Future study can measure body mass index because childhood adversity and D4 dopamine receptor gene are associated with obesity [31,32].

## 5. Conclusions

This study showed a specific pattern of interaction between mothers’ basic temperament traits (negative affectivity and effortful control) and genetic markers in their assessment of children’s emotional and behavioral problems. In particular, dopamine D4 receptor gene and children’s age at adoption are two moderators in the association in which mothers’ temperament was affecting their evaluation of their children’s emotional and behavioral problems. Beyond the acknowledged data on the importance of age at adoption for children’s likelihood of showing behavioral problems [33], our results suggest that temperamental and neurobiological markers do matter in adoptive mothers’ report on them. Despite the limitations mentioned, these findings may help in better understanding the mechanisms involved in adoptive parenting and, last but not least, in benefiting from the new directions offered by the promising research field of behavioral genetics of parenting in contributing to family mental health policies [16,17,18,19,20,21,22,23,24,25,26].

## Figures and Tables

**Table 1:** Demographic characteristics of adopted children and mothers.

	%	Mean (SD)	Range
Age at assessment (months)		43 (16.1)	14–75
Age at adoption (months)		33 (17.1)	1–68
Gender	Male: 58		
Mothers’ age (years)		42.7 (3.8)	34–51
SES ^1^		31.1 (6.9)	16.5–48.5
Length		11.2 (3.7)	3–20

^1^ SES = Socioeconomic status. Our sample comprised of mainly middle class Italian families.

**Table 2:** Zero-order correlations for study variables.

Variables	1	2	3	4	5	6
1. Child Total Problems	-	0.26 *	−0.25 *	0.22 *	0.54 ***	−0.33 **
2. Mother Temperament-NA		-	−0.45 ***	0.28 **	0.25 ^i^	−0.10
3. Mother Temperament-EC			-	0.04	−0.16	−0.17
4. Age at Adoption				-	0.23	0.06
5. Child Temperament-NA ^1^					-	0.01
6. Child Temperament-EC ^1^						-

*** *p* < 0.001, ** *p* < 0.01, * *p* < 0.05, ⁱ *p* = 0.07; ¹ *N* = 53.
